# Comparison of the effects of intravenous Dexketoprofen Trometamol versus Paracetamol on postoperative analgesia in patients undergoing Septoplasty: A randomised double-blind clinical trial

**DOI:** 10.12669/pjms.343.13804

**Published:** 2018

**Authors:** Caliskan E, Sener M, Kipri M, Yilmaz I, Aribogan A

**Affiliations:** 1 Caliskan E. Department of Anesthesiology and Reanimation, Baskent University Faculty of Medicine, Ankara, Turkey; 2 Sener M. Department of Anesthesiology and Reanimation, Baskent University Faculty of Medicine, Ankara, Turkey; 3 Kipri M. Department of Anesthesiology and Reanimation, Baskent University Faculty of Medicine, Ankara, Turkey; 4 Yilmaz I. Department of Otolaryngology, Baskent University Faculty of Medicine, Ankara, Turkey; 5 Aribogan A. Department of Anesthesiology and Reanimation, Baskent University Faculty of Medicine, Ankara, Turkey

**Keywords:** Dexketoprofen, Septoplasty, Postoperative pain, Paracetamol

## Abstract

**Background and Objective:**

Septoplasty operations are associated with moderate to severe postoperative pain which has unfavourable effects on patient’s recovery and postoperative outcome. The aim of this study was to compare effects of intravenous paracetamol and dexketoprofen on postoperative analgesia, tramadol consumption and side effects after septoplasty.

**Methods:**

In total 72 patients (aged 18–65 years) who had undergone an elective septoplasty from August 2013 to January 2015 in Baskent University Faculty of Medicine, in the Department of Anesthesiology and Reanimation Clinics were included in this study. The patients were randomised into one of two groups: those who received intravenous paracetamol; and those who received intravenous dexketoprofen. All patients were treated with tramadol for 24 hour postoperatively. The primary endpoint was pain intensity as measured by a visual analogue scale (VAS). Tramadol consumption and drug related side effects were also recorded.

**Results:**

The pain scores in the dexketoprofen group were significantly lower at recovery, 15 and 30 minutes and two hour (p< 0.05). The pain scores had no difference at other time points. Tramadol consumption in the recovery period was significantly lower in the dexketoprofen group, but cumulative tramadol consumption did not differ between the groups. The incidence of nausea was lower but not statistically significant in the dexketoprofen group at 15 and 30 minutes and two hour.

**Conclusions:**

Compared with paracetamol, preemptive dexketoprofen is associated with lower VAS scores and tramadol consumption in the early postoperative period after septoplasty. However, the cumulative tramadol consumption did not significantly differ between the groups.

## INTRODUCTION

Effective management of postoperative pain is main challenging for anaesthesiologists and clinicians despite impressive advances in analgesic drugs and techniques. One of the most important factors influencing patient safety, comfort and early discharge is effective postoperative pain control.^1^

Opioids are analgesic agents with proven efficacy and their administration has been a conventional method of pain control for many years. However, large bolus doses of potent opioids are now being used less frequently because of dose-dependent side effects and increased incidence of opioid base postoperative complications.^2^ In addition to these complications, inadequate treatment of pain is another cause of long-term hospitalisation, unexpected hospital admissions after surgery and increase health care costs.^3^ Considering these side effects, the use of balance multimodal analgesic protocols involving smaller doses of opioids in combination with non-opioid analgesic drugs (such as Nonsteroidal anti-inflammatory drugs [NSAID_S_], paracetamol, and local anaesthetics) has become a widely accepted approach both during and after surgery.^4^,^5^

Newly improved agents are used in clinical practice for the treatment of moderate and severe pain when administered orally, intramusculary, and intravenously.^6^ In recent years, two such drugs investigated in previous studies intravenous forms of paracetamol^7^ and dexketoprofen^8^ have played an important role in postoperative pain relief in the clinical setting. Paracetamol is among the most commonly used analgesic agents because of its safety and favourable side effects in postoperative pain management. Previous studies have shown that, paracetamol allows for decreased opioid consumption after different surgical procedures.^7^,^9^

Dexketoprofen trometamol is an active enantiomer of racemic ketoprofen and, centrally acting NSAID. Like other nonsteroidal agents, dexketoprofen has analgesic, anti-pyretic and anti-inflammatory properties.^10^ It’s advantages can be listed as faster onset of action and more potency and less gastrointestinal side effects when compared to ketoprofen and other NSAID’s. Although intravenous dexketoprofen trometamol has been used and compared with paracetamol in postoperative pain treatment in different surgeries, there are not enough studies on dexketoprofen paracetamol and opioid combination in patient controlled analgesia technique.

In present study, our primary endpoint was to compare the analgesic efficacy and side effect profile of dexketoprofen trometamol and paracetamol in patients with septoplasty when they were combined with intravenous tramadol patient controlled analgesia.

## METHODS

This study was approved by the Baskent University Institutional Ethics Committee (Project no. KA13/100) and Trial registration: Clinical Trials.gov Identifier: NCT02137395. After providing written informed consent, in 72 patients (American Society of Anaesthesiologist (ASA) physical status: I-II; aged 18–65 years) who had undergone an elective septoplasty from August 2013 to January 2015 in Baskent University Faculty of Medicine Department of Anesthesiology Clinics were enrolled in this study. The exclusion criteria were a history of significant cardiac, pulmonary, hepatic or renal disease; hypersensitivity to the study medications, any disorder contraindicating administration of non-opioid analgesics, administration of any analgesics 24 hour prior to the study and pregnancy.

**Fig.1:**
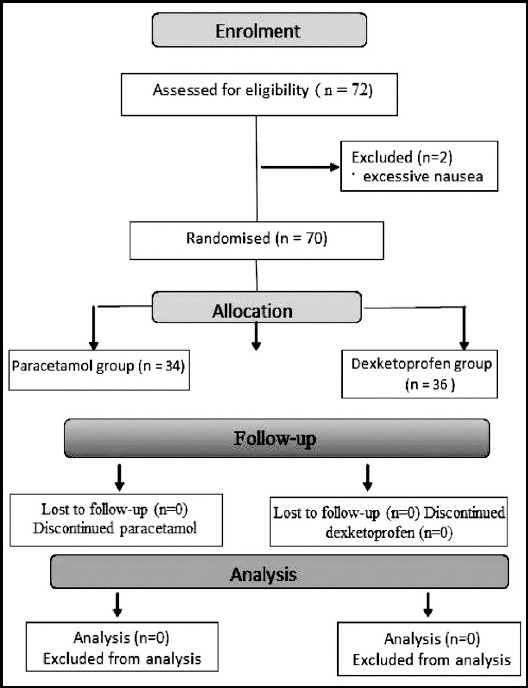
Flow chart of the study design. Administration of the study drug was started before the surgery and then repeated at 6-h (paracetamol) and 8-h (dexketoprofen) intervals for at least 24 h. Scores were then recorded at 24 h after surgery.

The patients were randomised into two parallel groups by a computer-generated random number list (http://www.randomization.com) (Fig.1). The assigned allocation group was written on a note placed inside a numbered envelope (one number corresponded to each patient enrolled); the envelope was then sealed.

### Paracetamol group

100 ml of an analgesic solution containing 1gr of paracetamol was administered as an infusion 15 minutes before the surgical incision.

### Dexketoprofen trometamol group

100 ml of an isotonic saline solution containing 50 mg dexketoprofen trometamol was administered as an infusion 15 minutes before the surgical incision.

At the preoperative visit, detailed instructions about how to use the PCA device and visual analogue scale (VAS) for pain assessment were provided to the patients by detail by blinded researcher. No premedication was given to the patients.

After admission to the operating room and standard anaesthesia monitoring, anaesthesia was induced with propofol (2 mg/kg), fentanyl (1μg/kg) and Rocuronium (0.1 mg/kg). After endotracheal intubation, anaesthesia was continued with 1.5% Sevoflurane and a 50% mixture of nitrous oxide and oxygen.

Postoperative pain intensity was evaluated using a VAS; (0 = no pain, 10 = worst pain imaginable) at recovery, 15 to 30 min, and 2, 4, 6, 12, and 24 h postoperatively. Intravenous tramadol PCA was used as a rescue analgesic in both groups. In the recovery room, patient’s VAS score was evaluated, additional 0.5 mg/kg of tramadol was intravenous administered if the score was >3 and intermittent intravenous 10 mg of tramadol was administered until the score was <3. The requirement of tramadol in this time period was recorded. After basal analgesia was supplied, PCA was given in 20-mg boluses with a lockout time of 20 min and a four hour limit arranged to 200 mg via PCA. Intravenous paracetamol and dexketoprofen were repeated every 6 and 8 h respectively, throughout the postoperative period.

Each time the VAS score was collected, the degree of sedation was determined according to a sedation score ranging from 0 to 2 (0 = alert, 1 = drowsy but arousable by voice, and 2 = very drowsy, but arousable by shaking.^11^

Patient satisfaction was measured using 5-point Likert Scale (very good, good, sufficient, poor, insufficient) at the end of the 24th hour.^12^ Additionally, the operation time, additional and cumulative tramadol consumption, postoperative adverse events and discharge from hospital were recorded.

The primary endpoint of this study was to compare the analgesic effects of intravenous dexketoprofen with those of paracetamol in combination with tramadol PCA during the first 24 hour following septoplasty. The secondary end-points were the effects of each PCA regimen Tramadol consumption and drug related side effects.

### Statistical analysis

A priori power analysis was performed based on the likely difference in the patients’ postoperative pain scores as evaluated by the VAS. The sample size was calculated by considering the results of our previous study of postoperative pain management, which was conducted in our otolaryngology clinic and in which the VAS was used.^13^ The results of our previous study showed, a mean VAS score of was 6.0±3.7 without additional analgesics in the postoperative period. Using these previous data, we performed a power analysis and determined that to detect a 45% reduction in the VAS scores of patients during the postoperative period after administration of analgesic drugs with 80% power at *a* = 0.05, we required a sample size of 30 patients per group. The analysis was performed by Power and Sample Size Calculation software (Vanderbilt University, Nashville, TN).

All statistical calculations were performed using SPSS for Windows, version 17.0 SPSS Inc., Chicago, IL). Differences in numerical variables between two groups were evaluated using a *t*-test or its nonparametric counterpart, the Mann–Whitney U-test depending on the distribution of variables. The Kolmogorov-Smirnov test was used to assess the normality of data distribution. Homogeneity of variance was calculated using Levene’s test and the Lilliefors tests. The chi-squared or Fisher’s exact test was used to evaluate the difference in qualitative differences. Data are expressed as means ± standard deviation or median (interquartile range). A p-value of < 0.05 was considered statistically significant.

## RESULTS

Seventy-two patients (36 patients in each group) were enrolled the study. During the follow-up period, two patients in the paracetamol group were excluded because of excessive nausea. Thus,70 patients (paracetamol group, 34 patients; dexketoprofen group, 36 patients) constituted the study population. There were no statistically significant differences with respect to age, sex, weight, discharge of hospital, duration of anaesthesia or surgery between the two groups (Table-I).

**Table-I:** Patients demographic data and surgical characteristics.

	Paracetamol (n=34)	Dexketoprofen (n=36)
Sex (male / female)	21/13	26/10
Age (y)	35± 13.3	32.3±11
Weight (kg)	76.2 ± 12.8	75.2±14.9
Height (cm)	168 ± 7	166 ± 8
Body mass index	27.1 ± 5.5	26.6 ± 5.3
ASA physical status (I/II)	30/4	28/8
Duration of surgery (min)	75 (55-90)	70 (60-87.5)
Discharge of hospital (hour)	23 ± 3.2	22.9 ± 2.5

Values are presented as n, mean ± standard deviation, and median (interquartile range).

ASA: American Society of Anesthesiologist.

**Fig.2:**
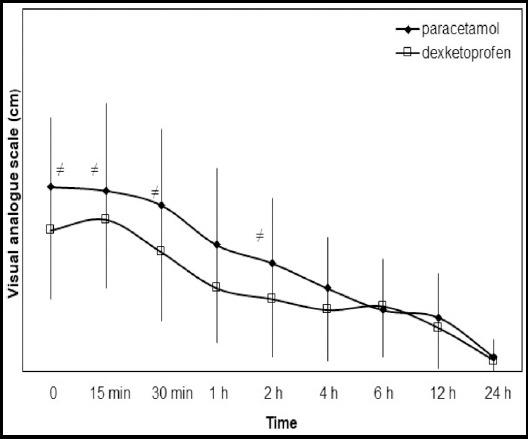
Visual analogue scale scores in the two groups at the indicated time intervals during the first 24h postoperatively. Values are presented as mean ± standard deviation. *p<0.05

Comparison of the groups at recovery, 15 and, 30 minutes, 1, 2, 4, 6, 12, and 24 hour showed that the VAS scores in the dexketoprofen group were significantly lower than those in the paracetamol group at recovery, 15 and, 30 minutes and two hour (p < 0.05) (Fig.2). There was no significant difference in pain scores at any other time points.

Tramadol consumption during the recovery period was higher in paracetamol group, but not significantly different between the paracetamol and dexketoprofen groups (Table-II). The total tramadol consumption was calculated by multiplying the number of deliveries at the end of the 24^th^ hour by the number of boluses (PCA demand and delivery values). There was also no significant difference in the total tramadol consumption, or, demand and delivery values between the two groups (Table-II).

**Table-II:** Postoperative tramadol consumption and PCA delivery and demand values.

	Paracetamol	Dexketoprofen	*p*
Duration of PACU (min)	39.1±10.4	32.3±6.3*	0.002*
Tramadol consumption at recovery (mg)	53.8±28.7	40±23.5	NS
Cumulative tramadol consumption (mg)	131.7±78.6	119±70	NS
PCA delivery (n)	6 (3-9)	5.5 (3.5-8.5)	NS
PCA demand (n)	9 (5-11)	7 (4-11.5)	NS

NS: Not significant,

* p<0.05. Values are presented as n, mean ± Standard deviation or median (interquartile range). PCA, patient-controlled analgesia; PACU, post-anesthesia care unit

During the postoperative 24 hour follow-up period, the two groups exhibited similar sedation scores, and the degree of sedation in all patients was generally determined to be mild during the recovery period and alert during the service follow-up period (p > 0.05).

Although the incidence of nausea was lower in dexketoprofen than paracetamol group during the postoperative in 24-h hours follow-up period, it was not statistically significant (p > 0.05). Thus, there is no statistically significant difference in vomiting and other side effects (Table-III).

**Table-III:** Incidence of side effects in each groups.

	Paracetamol	Dexketoprofen
Nausea	12 (35.2)	5 (13.8)
Vomiting	2 (5.8)	0
Gastric pain	1(2.9)	0
Gastrointestinal bleeding	1 (2.9)	0/36
Headache	3 (8.8)	1 (2.7)

Data are presented as n (%).

The overall degree patient’s satisfaction was determined to very good and good in both groups and there was a significant difference in patient satisfaction between the two groups. The number of patients who were very good was significantly higher in Group-II (29 patients) than in Group-I (6 patients).

## DISCUSSION

During the last 20 years, despite our increased knowledge of the pathophysiology of nociception and pharmacological alternatives for the treatment of pain remains a problem in many cases. Therefore, in recent years, multimodal or, “balanced” analgesic techniques with non-opioid analgesic drugs such as paracetamol and various NSAIDS in combination with lower doses of opioids are preferred for perioperative analgesia.^14^ Dexketoprofen and paracetamol, including in their intravenous forms are other analgesics that have been increasingly used in recent years too.^7^,^15^,^16^

In the present study, we analysed the effect of intravenous dexketoprofen trometamol and paracetamol administered at 24-h intervals on postoperative analgesic efficacy, tramadol consumption and side effects. Patients in dexketoprofen group had lower VAS scores in recovery, at 15 and, 30 minutes, and at two hour than patients in the paracetamol group; they also had lower consumption of tramadol in the recovery period than patients in the paracetamol group.

NSAIDs are widely used as supplemental analgesics, however, they should be carefully used because of their potential adverse effects including renal and gastrointestinal side effects, bone marrow suppression, and postoperative bleeding.^17^Dexketoprofen and paracetamol, including in their intravenous forms are other analgesics that have been increasingly used in recent years.^7^,^15^,^16^

Intravenous paracetamol is commonly used analgesic for acute postoperative pain management. Paracetamol exhibits central inhibition of cyclooxygenases and interact with seratoninergic system^18^ and does not include have the side effects seen with NSAIDs. The effects of intravenous paracetamol on postoperative pain have been previously studied in different surgeries and also evaluated and demonstrated opioid-sparing effects.^16^,^19^

Broadner et al.^19^ showed that the analgesic efficacy of paracetamol is not different from the efficacy of dipyrone and parecoxib when used to provide postoperative analgesia after various surgical procedures. Therewithal, Korkmaz et al.^20^ demonstrated that 1g IV pre-emptively paracetamol in addition to morphine PCA provided effective equivalent analgesia to 1g of metamizole after lumbar disc surgery.

In our study, we used 1g IV paracetamol and but, we could not show any reduction in pain scores and tramadol consumption compared with dexketoprofen. Ketoprofen is an NSAID with analgesic properties, but it also has severe gastrointestinal side effects. Dexketoprofen trometamol is an active enantiomer of racemic ketoprofen which has equivalent analgesic efficacy with less severe side effects than ketoprofen.^10^ The analgesic efficacy of oral or intramuscular dexketoprofen on moderate and severe pain have evaluated in previous studies^10^,^21^,^22^ and demonstrated lower VAS scores and opioid consumption in the dexketoprofen group than in the control group.^22^

In recent years, the parenteral formulation of dexketoprofen has been improved and now demonstrates adequate and safe analgesic properties and tolerable side effects in various surgical procedures.^18^ Ozer et al.^23^ compared the analgesic efficacy and side effect profile of preoperative and/or intraoperative intravenous administration of dexketoprofen with those in a control after septorhinoplasty. The dexketoprofen group exhibited significant analgesic efficacy with lower VAS scores, less severe side effects, and good patient complacence.

In literature, there are several studies which have compared the effects of intravenous paracetamol and dexketoprofen on postoperative pain.^16^ But, the number of studies use these drugs in combined with patient-controlled analgesia is more limited.^16^,^24^

Tunali et al.^16^ compared the analgesic effect of intravenous paracetamol with that of dexketoprofen on postoperative pain after lumbar disc surgery. This study showed that pain intensity was significantly lower with dexketoprofen as a supplemental analgesic to morphine PCA when compared with the control group during the first 24 hour postoperatively. In the same study, there was no difference in the pain scores when the dexketoprofen group was compared with the paracetamol group.

In our study, the VAS scores in the dexketoprofen group were significantly lower than those in the paracetamol group at recovery, 15 and, 30 min and 2 h (p < 0.05). This difference can be explained by having more patients in our study and by the difference in the type of surgery.

These results also indicate that dexketoprofen provides more effective analgesia in the early postoperative period after septoplasty than does paracetamol. The low consumption of opioids during the recovery period also supports these results.

Although, in the present study, we found that dexketoprofen reduced the intensity of pain in the early follow-up period and opioid requirement in recovery period, but cumulative tramadol consumption between two groups were not significantly different when used as a supplemental analgesic to tramadol PCA.

A possible explanation for these findings might be that the sample size in our study was not large enough to detect substle differences. Our power analysis was based on the assumption that 45 % decrease in pain score on a VAS clinically relevant and should be primary outcome measure, but this might be sufficiently large to detect tramadol consumption.

Postoperative nausea and vomiting is among the most common postoperative side effects following general anaesthesia. The addition of the NSAIDs to multimodal analgesia protocols, while reducing the side effects associated with opioids, can also cause gastrointestinal side effects of their own.^4^

In previous studies involving different types of surgery, dexketoprofen possessed good analgesic efficacy and a high tolerability profile with lower gastrointestinal side effects.

Yazar et al.^8^ compared dexketoprofen with a control drug in 60 patients who underwent lumbar disc surgery. The authors found that 50 mg intravenous dexketoprofen administered intraoperatively and at 12 hour postoperatively was an effective analgesic that decreased opioid consumption and opiod-related side effects. These findings also support the results of our study.

In our study, a supplemental analgesic used occurred more frequently in the paracetamol than dexketoprofen group. In addition to significantly lower pain scores and lower opiod consumption with dexketoprofen than paracetamol, we also found lower incidence of nausea in the dexketoprofen group, but without statistical significance. We believe that a statistical difference could be reached by increasing the sample size, which is a limiting factor in our study. Likewise, we believe that the high incidence of nausea in the paracetamol group can also be explained of the higher opioid dose for supplemental analgesia. Intravenous dexketoprofen given with tramadol PCA was better tolerated than paracetamol. The other side effects and sedation scores did not differ between two groups. In our study, the overall degree patient’s satisfaction was determined to very good and good in both groups. The number of patients who were very satisfied was significantly higher in dexketoprofen group which we thought have been associated with the lower incidence of nausea.

## CONCLUSION

The concept of preemptive analgesia remains an important research topic for postoperative pain relief. The results of our study show that 50 mg of dexketoprofen trometamol administered 15 minutes before the surgical incision and the during the first 24h postoperatively (every 8 h) is an effective analgesic that lowers pain scores and, decreases opioid consumption and opioid related side effects compared with paracetamol in patients undergoing septoplasty. Effective postoperative pain relief, the opioid sparing effect, and the low incidence of side effects with dexketoprofen may be one main reason to use this drug as an alternative to paracetamol. We feel that, further controlled studies comparing intravenous dexketoprofen with other NSAID_S_ and using different dose regimens are needed to determine the efficacy and safety profile of dexketoprofen.

### Author’s Contribution

**EC:** Conceived design and statistical analysis & preparation of manuscript.

**MS:** statistical analysis and prepare the analgesic solution.

**MK:** did data collection.
